# Associations Between 24 h Movement Behaviours and Cognitive Abilities in Slovak Adolescents: A Cross-Sectional Study

**DOI:** 10.3390/healthcare14030360

**Published:** 2026-01-30

**Authors:** Beata Ruzbarska, Lenka Hnidkova, Mojmir Trebunak, Erika Chovanova, Dalibor Dzugas, Peter Kacur

**Affiliations:** Faculty of Sport, University of Prešov, 17. Novembra 15, 080 01 Prešov, Slovakia; beata.ruzbarska@unipo.sk (B.R.); lenka.hnidkova@unipo.sk (L.H.); mojmir.trebunak@unipo.sk (M.T.); erika.chovanova@unipo.sk (E.C.); dalibor.dzugas@unipo.sk (D.D.)

**Keywords:** 24 h movement behaviours, adolescent cognition, physical activity, sleep, sedentary behaviour, accelerometry, PAQ-A

## Abstract

Background: Twenty-four-hour movement behaviours (physical activity, sedentary time, and sleep) may be associated with adolescent cognitive performance, but evidence from Central/Eastern Europe is limited. Methods: A total of 82 Slovak adolescents (15–19 years) completed tests of IQ, attention, and visual memory. Participants wore a wrist accelerometer 24/7 for seven consecutive days (processed in GGIR v3.0–3). Moderate-to-vigorous physical activity (MVPA), total sedentary time, and sleep duration were derived from accelerometry; physical activity was also self-reported using the Physical Activity Questionnaire for Adolescents (PAQ-A). Non-parametric tests and Spearman correlations were applied; sleep × MVPA interaction models (robust HC3 standard errors) were adjusted for age and sex. Results: MVPA was low (median 32.9 min/day; 11% met ≥60 min/day), while sedentary time was high (median 652.6 min/day). Associations between movement behaviours and cognition were generally small, and no sleep × MVPA interaction effects were observed. The PAQ-A overestimated device-based MVPA (mean bias +1.68 units; 95% limits of agreement +1.10 to +2.25), with greater overestimation in girls and older adolescents. Conclusions: In this convenience sample, 24 h movement patterns were suboptimal, and their associations with cognition were modest and exploratory. Larger longitudinal studies are needed to confirm these findings.

## 1. Introduction

The relationship between 24 h movement behaviours (24-HMBs)—physical activity (PA), sedentary behaviour (SB), and sleep (SL)—and cognitive development in adolescents has garnered increasing attention. Cognitive functions such as attention, memory, and higher-order executive processes undergo substantial maturation during adolescence and may be sensitive to lifestyle-related behaviours [1,2]. Because these behaviours jointly occupy the finite 24 h day, examining PA, SB, and sleep within an integrated framework may provide a more ecologically valid perspective than studying each behaviour in isolation.

A substantial body of evidence links higher levels of PA, particularly moderate-to-vigorous physical activity (MVPA), with better cognitive performance in youth, especially in executive domains such as inhibitory control, working memory, and cognitive flexibility [3,4]. However, findings vary by cognitive domain, measurement approach, and the context in which PA is accumulated (e.g., structured vs. incidental activity) [5,6,7]. Sleep duration and quality are also consistently related to cognitive outcomes in adolescence. Meta-analytic and observational evidence suggests that insufficient sleep is associated with poorer attention, learning, and academic outcomes, whereas a mid-range sleep duration may be most favourable [1,2,8,9,10,11].

In contrast, associations between SB and cognition appear more nuanced and may depend on the type and content of sedentary activities (e.g., screen-based vs. cognitively enriching sedentary behaviours) [12,13,14]. High screen time has been linked to attentional difficulties and neurocognitive indicators consistent with reduced cognitive efficiency [13,15,16], while some non-screen sedentary behaviours may be neutral or context-dependent [12,13,14]. These mixed findings highlight the need for careful operationalisation of SB and for transparent reporting of SB outcomes.

Emerging studies also suggest that PA and sleep may interact. Adolescents who combine adequate sleep with higher PA may demonstrate better cognitive performance than those with favourable levels in only one behaviour, and sleep may moderate associations between PA and cognitive outcomes [17,18]. Nevertheless, the literature remains limited by frequent single-behaviour approaches, inconsistent operational definitions, and reliance on self-reported measures, which may overestimate PA—particularly among younger adolescents and girls [19,20]. Objective assessment using accelerometry can improve measurement validity and enable a more detailed description of accumulation patterns across the day [21,22].

Critical regional gaps also persist. Central and Eastern European populations, including Slovak adolescents, remain underrepresented in studies that combine objectively measured 24-HMBs with multi-domain cognitive assessments [23]. Moreover, many studies focus on broad academic indicators or global intelligence scores, placing less emphasis on distinct domains such as attentional control and visual memory, which may exhibit differential sensitivity to movement behaviours [14,24].

In response to these gaps, the present study investigates how three 24-HMBs, physical activity (PA), sedentary behaviour (SB), and sleep, are associated with cognitive functions (IQ, attention, and visual memory) in Slovak adolescents aged 15–19 years. In addition, the study examines the validity of self-reported PA using the PAQ-A compared to accelerometer data. It explores the interaction between moderate-to-vigorous physical activity (MVPA) and sleep on cognitive performance. The study addresses four main research questions: (1) How do MVPA, total sedentary time (minutes/day), and sleep duration differ by sex and age, and what proportion meet MVPA (≥60 min/day) and sleep (≥8 h/night) recommendations? (2) How are specific movement behaviours (sleep duration/timing, MVPA, sedentary time, and selected accumulation metrics) associated with IQ, attention, and visual memory? (3) Does sleep duration moderate the association between MVPA and cognitive outcomes (sleep × MVPA interaction)? (4) How accurately does self-reported PA (PAQ-A) reflect accelerometer-derived MVPA, and does disagreement differ by sex or age? We hypothesised that more favourable 24 h movement behaviours (higher MVPA, longer sleep duration, and lower total sedentary time) would be associated with better cognitive performance (IQ, attention, and visual memory) in Slovak adolescents.

## 2. Materials and Methods

### 2.1. Study Design and Participants

This cross-sectional study included 82 adolescents aged 15 to 19 years (mean age = 16.67 years, SD = 1.07). The sample comprised 32 males (M = 16.72 years, SD = 1.13) and 50 females (M = 16.64 years, SD = 1.05). Participants were recruited from secondary schools during a typical academic year using convenience sampling. Participation was voluntary, and no financial incentives were offered. Adolescents were eligible if they were enrolled in regular schooling, were able to participate in usual daily activities, and did not report any acute illness on the day of testing. Participants were stratified by age as follows: *n* = 12 were 15 years, *n* = 32 were 16 years, *n* = 24 were 17 years, and *n* = 14 were 18–19 years. Only participants with complete cognitive testing, a valid PAQ-A, and sufficient accelerometer data were included in the analysis.

### 2.2. Study Size

The study used a convenience sample based on feasibility and school participation. Given the final sample size (*n* = 82), analyses were interpreted cautiously and considered hypothesis-generating. More minor effects and interaction effects typically require larger samples; therefore, moderation analyses were treated as exploratory. No a priori sample size calculation was performed because recruitment was constrained by school participation and feasibility. Thus, analyses were interpreted as exploratory. As a sensitivity benchmark, with *n* = 82 and α = 0.05 (two-tailed), the study has ~80% power to detect correlations of approximately |ρ| ≥ 0.31; more minor effects and interaction terms are likely underpowered.

### 2.3. Cognitive Assessment

Sustained attention and concentration were assessed using the Kučera Attention Concentration Test [25]. This cancellation task requires participants to cross out specific target symbols among visually similar distractors within a fixed time limit. The test yields indices of processing speed, accuracy, error rate, and psychomotor tempo and has been widely used in Czech and Slovak school-aged and adolescent populations. Contemporary psychodiagnostic manuals provide norms and interpretive guidelines for the use of these instruments in older children and adolescents [26]. In the present study, we used the total number of correctly marked items and the number of errors as primary attention outcomes, with higher correct scores indicating better performance.

General intellectual ability was measured using the Test of Intellectual Potential (TIP) developed by Říčan [27]. The TIP is a nonverbal reasoning test that assesses fluid intelligence through 29 figural series, in which participants identify the missing element based on the underlying rules governing the sequence. Raw scores can be converted to IQ or standardised scores using age-appropriate norms, which are available for older school-age children and adolescents and are still recommended as part of cognitive test batteries in Central European settings [26]. In this study, age-standardised IQ scores derived from the TIP served as the primary indicator of general intellectual ability.

Short-term visual memory was assessed with the Meili Visual Memory Test [28,29]. Participants view a matrix of pictorial stimuli for 1 min, then have 5 min to freely recall and write down as many items as they can remember. The total number of correctly recalled items reflects the capacity of primary visual memory and the predominant visual memory style. Normative data and administration guidelines for adolescents are provided in recent Slovak methodological materials [29]. Higher scores indicate better short-term visual memory performance.

### 2.4. Assessment of 24 h Movement Behaviours

Objective physical activity (PA), sedentary behaviour (SB), and sleep were assessed using wrist-worn triaxial accelerometers (ActiGraph wGT3X-BT, ActiGraph LLC, Pensacola, FL, USA). Participants wore the device on the non-dominant wrist for seven consecutive days, 24 h/day, during a typical school week, removing it only for water-based activities. Devices were initialised in accordance with the manufacturer’s recommendations. Raw acceleration data were processed in R using the GGIR package (v3.0–3) [30], which performs auto-calibration, detection of non-wear time, and derivation of time spent in different intensity categories. Age-specific cut-points and processing thresholds for youth were based on Hildebrand et al. [31] and related work on wrist-worn accelerometry in children and adolescents, allowing the extraction of time spent in sleep, SB, light PA, moderate PA, vigorous PA, and moderate-to-vigorous PA (MVPA). Within GGIR, non-wear and invalid segments were detected and handled using the package’s standard procedures. Days with ≥16 h of wear time were considered valid. Participants with clearly insufficient monitoring (<16 h/day) were excluded. Across the final analytic sample (*n* = 82), the average number of valid days was 4.9 ± 2.0 (median 5 [IQR 4–7], range 1–7), indicating that most participants provided sufficient accelerometer data for analysis. Days that did not meet the wear-time criterion were treated as invalid and excluded from daily summaries. Non-wear time was detected using GGIR default algorithms; no manual edits were performed. Daily variables were calculated as the mean across valid days.

In addition to total time, bout-based and peak activity indices were derived to capture the accumulation and intensity pattern of movement behaviours. Bouts were defined as consecutive epochs (10 s sampling interval) within the same intensity category, allowing ≤1 epoch below threshold between segments. The distribution of bouts was classified into 0–1 min, 1–10 min, and 10–30 min durations for sedentary behaviour and light activity, and into 5–10 min durations for MVPA. Peak activity metrics represented the mean acceleration within the most active 30, 60 min, 5 h, and 10 h periods of the day, and the least active 5 h period (“L5”). These indices characterise daily movement rhythms and activity fragmentation.

Self-reported PA was assessed using the Physical Activity Questionnaire for Adolescents (PAQ-A) [32]. The PAQ-A is a self-administered, 7-day recall instrument designed for 14–19-year-olds that captures general levels of MVPA during the school year. It consists of 9 items rated on a 5-point Likert scale, covering activities at school, during physical education, in leisure time, and on weekends. The PAQ-A has demonstrated acceptable internal consistency (Cronbach’s α ≈ 0.75–0.82) and moderate correlations with objective MVPA in adolescent samples [32,33].

### 2.5. Procedure

Data collection took place during regular school weeks across all age groups. Testing sessions were scheduled during school hours in collaboration with school administrators. After receiving study information, participants (and their legal guardians for minors) provided written informed consent. Cognitive assessments were administered first, in small groups, in a quiet classroom environment under standardised conditions by trained research staff (a psychologist or trained graduate students). The order of tests was kept consistent across classes to reduce order effects. Immediately after the cognitive tests, participants completed the PAQ-A, with researchers available to clarify any uncertainties. At the end of the classroom session, accelerometers were distributed and fitted on the non-dominant wrist. Participants received written and oral instructions to wear the device continuously for seven days (including nights), removing it only for bathing, showering, or swimming. After the monitoring period, devices were collected at the school, and the data were downloaded, anonymised, and checked for completeness and signal quality before analysis.

### 2.6. Statistical Analysis

Normality of continuous variables was assessed using visual inspection (histograms and Q–Q plots) and the Shapiro–Wilk test. Several variables deviated significantly from normality (Shapiro–Wilk *p* < 0.05); therefore, non-parametric methods were used where appropriate. Four specific research questions (RQ1–RQ4) guided the analytical procedures, each aligned with a distinct statistical approach. To address RQ1, the sample was compared by sex (male vs. female) and age group (15, 16, 17, 18–19 years). The Mann–Whitney U test was used to identify differences in key movement behaviour variables: time spent in MVPA, SB, and total sleep duration. Distribution plots were used to examine adherence to thresholds for MVPA (≥60 min/day) and sleep duration (≥8 h/night). Because there is no universal consensus on a time-based threshold for total accelerometer-derived sedentary time in adolescents, sedentary time was reported descriptively (minutes/day) without guideline-based adherence classification. Effect sizes (r) were reported to interpret the magnitude of group differences.

To investigate RQ2, bivariate associations between movement behaviours (MVPA, SB, sleep duration and timing, bouted variables, and peak activity indices) and cognitive scores (IQ from the TIP, accuracy and errors from the Attention Concentration Test, and visual memory recall) were examined using Spearman’s rank-order correlation (ρ). A non-parametric approach was selected due to violations of normality in multiple variables. Correlation coefficients were interpreted based on both statistical significance (*p* < 0.05) and practical relevance (ρ ≥ 0.30 considered moderate).

For RQ3, we tested whether sleep duration moderates the association between MVPA and cognitive outcomes using multiple linear regression models with an interaction term (sleep × MVPA). Sleep duration was expressed in hours/night and MVPA in 10 min units (min/day ÷ 10), mean-centred, and the product term was computed from the centred variables. Models were adjusted for age and sex. Given the sample size, interaction effects were interpreted cautiously and treated as exploratory; robust standard errors (HC3) were used. Model assumptions were checked visually. These models were used to avoid small cell sizes in subgroups and were considered exploratory.

To address RQ4, the PAQ-A summary score and accelerometer-derived MVPA (min/day) were compared using the Bland–Altman method, providing the mean difference (bias) and 95% limits of agreement (mean difference ± 1.96 SD). In addition, MVPA estimates from the PAQ-A and accelerometry, as well as the magnitude of overestimation, were compared across age categories using the Kruskal–Wallis H test, followed by post hoc Mann–Whitney U tests where appropriate. Accelerometer data processing was performed in R using GGIR (v3.0–3). Statistical analyses were performed in Python 3.11.9 using pandas (2.2.2), SciPy (1.13.1), and statsmodels (0.14.2). Statistical significance was set at *p* < 0.05 (two-tailed), with effect sizes reported alongside *p*-values where applicable.

### 2.7. Use of Generative Artificial Intelligence

Generative artificial intelligence tools (ChatGPT (OpenAI, version 5.1), SciSpace AI (web-based platform; https://scispace.com; accessed 29 October 2025)) were used solely to improve the clarity of the manuscript text and to generate partial graphic interpretations. All AI-assisted text was carefully checked and revised by the authors, who take full responsibility for the final content.

## 3. Results

### 3.1. Adherence to 24-H Movement Behaviour Guidelines and Sex-Based Differences in Movement Behaviours

Overall adherence to MVPA and sleep recommendations was low in the total adolescent sample (Table 1). Total sedentary time was high (median 652.6 min/day; IQR 530.4–706.5), corresponding to approximately 10.9 h/day (Supplementary Figure S1b). Sleep duration was similarly suboptimal. The median sleep duration was 433 min per night (~7.2 h), and most participants did not meet the minimum recommendation of 480 min (8 h) (Supplementary Figure S1c). The peak of the distribution ranged from 400 to 460 min (6.5 to 7.5 h). Nearly two-fifths (39%) experienced short sleep (≤420 min), while 48.8% achieved adequate sleep and 12.2% reported long sleep durations. Girls showed higher compliance with sleep recommendations (56%) than boys (37.5%), suggesting sex-related differences in nightly recovery patterns. Regarding moderate-to-vigorous physical activity (MVPA), the distribution was left-skewed (Supplementary Figure S1a), with most participants engaging in 10–40 min per day. The median daily MVPA was 32.9 min, and only 11% of the total sample met the ≥60 min per day guideline. The lowest compliance was observed among 18–19-year-olds (7.1%), and no 15-year-olds met the MVPA recommendation. Overall, adolescents exhibited patterns of insufficient physical activity, prolonged sedentary time, and inadequate sleep, underscoring the need to address 24 h movement imbalances.

In sex-stratified comparisons, girls accumulated more MVPA than boys (median 37.7 [IQR 22.9–54.1] vs. 18.9 [9.1–40.5] min/day; U = 506.0, *p* = 0.005, r = 0.308) and also more total sedentary time (666.4 [603.0–706.9] vs. 587.7 [196.9–686.9] min/day; U = 565.0, *p* = 0.026, r = 0.246). Detailed sex differences in bout- and peak-based activity metrics are provided in Supplementary Table S1.

### 3.2. Associations Between Movement Behaviours and Cognitive Performance

Spearman correlation analysis identified several significant associations between sleep and activity variables and cognitive outcomes. Later sleep onset time was positively associated with estimated IQ (ρ = 0.24, *p* = 0.031), as was later wake-up time (ρ = 0.30, *p* = 0.006). Longer deep sleep duration was positively associated with estimated IQ (ρ = 0.23, *p* = 0.039) and attention performance (responses: ρ = 0.22, *p* = 0.048; correct responses: ρ = 0.24, *p* = 0.033). In contrast, total sleep duration was negatively associated with memory scores (ρ = −0.24, *p* = 0.030). With respect to activity, a greater number of sedentary bouts lasting 1–10 min was negatively correlated with the number of incorrect attention responses. MVPA accumulated in 5–10 min bouts was inversely associated with estimated IQ. Higher values in the least active 5 h period were also negatively associated with both estimated IQ and memory scores. Complete correlation coefficients and *p*-values are provided in Supplementary Figure S2.

### 3.3. Combined Role of Sleep and MVPA (Moderation Models)

To avoid unstable inference from tiny profile groups, we examined the combined role of sleep and MVPA using a continuous moderation model (sleep × MVPA interaction), adjusted for age and sex. The sleep × MVPA interaction was not supported (IQ: B = 0.283, 95% CI [−0.300; 0.865], *p* = 0.342; attention: B = 0.107, 95% CI [−1.075; 1.290], *p* = 0.859; memory: B = 0.001, 95% CI [−0.222; 0.224], *p* = 0.991). Adding the interaction term resulted in negligible changes in explained variance (ΔR^2^ = 0.014 for IQ; 0.001 for attention; ~0.000 for memory). Detailed regression estimates are provided in Table 2.

### 3.4. Agreement Between PAQ-A and Objective MVPA Measures

**Figure 1:**
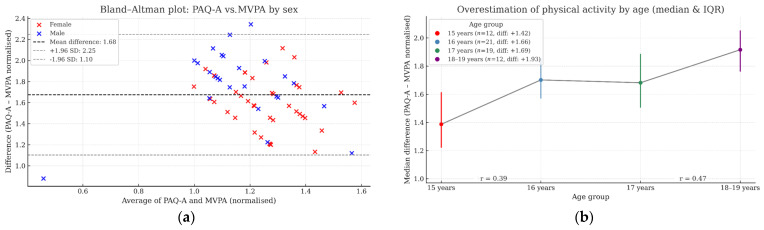
Agreement between PAQ-A and accelerometer-derived MVPA. (**a**) Bland–Altman plot comparing PAQ-A scores with accelerometer-based MVPA. Each point represents an individual participant. The solid line indicates the mean difference between PAQ-A and accelerometer-based MVPA (+1.68 units), and the dashed lines represent the 95% limits of agreement (+1.11 to +2.25). (**b**) Age-related overestimation of MVPA by PAQ-A. Bars show mean differences between PAQ-A scores and accelerometer-derived MVPA for each age group (15, 16, 17, 18–19 years). Positive values indicate overestimation of MVPA.

The comparison of self-reported PAQ-A scores with accelerometer-based MVPA indicated a systematic overestimation. Bland–Altman analysis (Figure 1a) revealed a mean difference of +1.68 units (SD = 0.29), with 95% limits of agreement from +1.10 to +2.25. Females showed greater overestimation (mean = +1.76) than males (mean = +1.57). This difference was statistically significant (U = 427.0, *p* = 0.008, r = 0.36). Age-based analysis (Figure 1b) showed a progressive increase in overestimation: 15 years: +1.42; 16 years: +1.66; 17 years: +1.69; 18–19 years: +1.93. A Kruskal–Wallis test confirmed significant differences (H(3) = 18.17, *p* = 0.0004). Post hoc tests revealed significant differences between 15–16 (*p* = 0.01, r = 0.39) and 17–18/19-year-olds (*p* = 0.003, r = 0.47). These findings suggest that overestimation of MVPA increases with age and is more pronounced among females, underscoring the importance of accounting for sex and age when interpreting self-reported physical activity in adolescence.

## 4. Discussion

### 4.1. Behavioural Compliance and Sex- and Age-Based Differences

The present findings underscore a critical issue in adolescent health: the low adherence to the MVPA and sleep recommendations across the sample. Only 11% of participants met the recommended threshold of 60 min of daily MVPA, while less than a third achieved sufficient sleep duration (≥8 h per night). Sedentary time was high, corresponding to approximately 10.9 h/day. This general pattern of non-compliance provides a foundational context for interpreting the cognitive outcomes reported in this study.

Consistent with global trends [35], these results reflect a growing concern regarding declines in physical activity, suboptimal sleep, and prolonged sedentary time among youth. A recent meta-analysis found that only about 7.1% of children and adolescents globally meet all three movement behaviour guidelines [36]. Subgroups such as those with ADHD, disabilities, or obesity report even lower compliance [37], compounding their vulnerability to cognitive and psychosocial difficulties.

Importantly, sex-based differences in movement behaviours were observed in both our data and the recent literature. Boys tend to engage in more vigorous physical activity and are more likely to meet MVPA guidelines [38]. At the same time, girls typically spend more time in sedentary activities, particularly during morning or school-related periods [39]. Our data confirmed that females reported longer durations across multiple sedentary bout categories and higher light activity levels, suggesting more fragmented movement throughout the day.

Age-related patterns indicated generally poorer adherence in mid-to-late adolescence, with 18–19-year-olds showing the lowest MVPA compliance, 15-year-olds showing the lowest MVPA adherence, while sedentary time remained high across age groups, and 16-year-olds showed the lowest proportion achieving adequate sleep. These findings align with evidence of age-related declines in MVPA and increases in sedentary time throughout adolescence [40,41]. Although some studies report slightly longer sleep duration in late adolescence, this may coexist with increased sleep fragmentation and delayed bedtimes, negatively affecting sleep quality [42,43].

These behavioural trends have meaningful implications for interpreting the results of RQ2–RQ4. Restricted variability in behaviours, the modest sample size, and measurement noise may have limited power to detect small associations and particularly interaction effects (sleep × MVPA). Therefore, the findings, particularly those related to the synergistic impacts, should be interpreted cautiously and treated as exploratory. Furthermore, any associations observed must be interpreted within the context of a behaviourally suboptimal sample, in which cognitive benefits associated with healthy 24 h behaviours may be attenuated or obscured.

### 4.2. Associations Between Movement Behaviours and Cognitive Performance

In this convenience sample, associations between 24 h movement behaviours and cognitive performance were generally small and domain-specific, suggesting that features of the daily movement–sleep profile may relate to selected cognitive outcomes in adolescents. Given the cross-sectional design, modest sample size, and number of metrics tested, these results should be interpreted as exploratory and hypothesis-generating rather than as evidence of a causal effect of daily behaviours on cognition.

Sleep characteristics showed the most consistent associations with cognitive outcomes. Later sleep onset and wake-up times were positively associated with estimated IQ in our data, while longer deep sleep was modestly associated with higher IQ and better attention performance. Although sleep timing and deep sleep are commonly considered important for cognitive regulation in adolescence [1,8,44], the direction of timing-related associations may also reflect contextual factors (e.g., chronotype, school schedules, or unmeasured socioenvironmental influences) and should therefore be interpreted cautiously. Sleep also contributes to memory consolidation, and insufficient or disrupted sleep has been linked to poorer long-term memory and academic outcomes in adolescents [2,44].

Interestingly, MVPA accumulated in short (5–10 min) bouts was inversely associated with estimated IQ (ρ = –0.24), and this association should be interpreted cautiously. One possibility is that short MVPA bouts reflect incidental or fragmented activity (e.g., brief transitions or non-purposeful movement) rather than structured exercise typically associated with cognitive benefits. Alternatively, the finding may reflect residual confounding (e.g., academic track, socioeconomic factors, chronotype, or organised sport participation) or chance variation due to multiple testing in a modest convenience sample. Future studies should distinguish between structured and incidental MVPA and test whether longer, sustained MVPA bouts are associated with different cognitive outcomes. This unexpected direction warrants replication and should be viewed primarily as a signal for further investigation rather than a definitive pattern [45,46]. In addition, mechanistic pathways involving physical fitness are plausible, but physical fitness was not assessed in the present study and should be measured explicitly in future research.

Regarding sedentary behaviour, the results suggest that not only total sedentary time but also the pattern of accumulation (i.e., fragmentation vs. prolonged uninterrupted periods) may be relevant for selected cognitive outcomes. However, these associations should be interpreted cautiously, given the modest sample size, multiple comparisons, and potential unmeasured confounding (e.g., school-related sitting, screen-based learning, and socioeconomic context). Future work should examine sedentary behaviour alongside sleep timing and structured physical activity within a unified 24 h framework, using longitudinal or intervention designs to clarify directionality and reduce the risk of spurious findings.

### 4.3. Synergic Effect of Sleep and MVPA on Cognitive Performance

Using a continuous moderation model, we found no clear evidence that sleep duration moderates the association between MVPA and cognitive outcomes (i.e., the sleep × MVPA interaction was not supported for IQ, attention, or memory). This null finding may reflect limited power to detect interaction effects in a sample of 82 adolescents, restricted variability in behaviours, and measurement noise. Therefore, combined (interactive) effects of sleep and MVPA on cognition should be considered exploratory in this study and warrant confirmation in larger longitudinal samples.

Although our data did not support a sleep × MVPA interaction, previous studies indicate that MVPA may attenuate the negative cognitive correlates of inadequate sleep. For example, adolescents with higher MVPA levels have shown better executive functioning and academic performance despite suboptimal sleep duration [46]. Similarly, participation in cognitively demanding or vigorous physical activities has been associated with improved neurocognitive functioning, even in the presence of lifestyle risk factors [13,47].

The compensatory role of MVPA has also been examined in relation to recreational screen time, in which higher physical activity levels attenuated the adverse effects of prolonged sedentary behaviour on attention and academic outcomes [46]. This suggests that MVPA may act as a behavioural buffer under suboptimal lifestyle conditions. However, such buffering effects were not clearly observable in our moderation models, which again supports interpreting the present findings as hypothesis-generating.

Despite these promising findings, it is essential to note that physical activity cannot fully substitute the cognitive benefits of adequate sleep. Both behaviours appear to operate through distinct yet complementary neurobiological pathways. While MVPA improves cerebral blood flow, neurogenesis, and executive control [47], sleep—particularly slow-wave sleep—is essential for memory consolidation and emotional regulation [8,44].

Therefore, health interventions should not focus on promoting a single behaviour in isolation but rather adopt an integrative approach that supports adequate sleep and daily MVPA. Although we did not observe a statistically significant interaction in this sample, the low proportion of adolescents meeting both recommendations underscores the practical importance of targeting both behaviours in future interventions and longitudinal studies.

### 4.4. Validity of Self-Reported Physical Activity (PAQ-A) in Adolescents

The present findings reveal a systematic overestimation of moderate-to-vigorous physical activity (MVPA) when self-reported using the PAQ-A compared with accelerometer-based measurements. The observed mean difference and narrow confidence intervals in the Bland–Altman analysis suggest that the PAQ-A tends to inflate physical activity levels across the sample. This trend is consistent with previous research indicating the moderate criterion validity of the PAQ-A when validated against objective tools, such as accelerometers [48,49]. These discrepancies may arise from the PAQ-A’s recall-based nature and the cognitive demands placed on adolescents to summarise activity across multiple contexts.

Sex-based differences further underscore the variability in reporting accuracy. Female participants exhibited significantly greater overestimation of MVPA than males, consistent with evidence that self-reported physical activity among adolescents is often overestimated. It may be particularly susceptible to social desirability bias among females [50,51]. While boys typically engage in more MVPA [38], their self-reported MVPA appears more closely aligned with accelerometer-based data. This sex-specific bias underscores the importance of stratified analysis in studies using self-report instruments.

Age-related trends were also observed, with older adolescents (18–19 years) showing the highest levels of overestimation. Although the previous literature suggests that cognitive maturity may enhance the accuracy of self-reports among older youth [49], our findings do not support this assumption. Instead, the increase in sedentary behaviours with age and possible disengagement from structured physical activities may complicate accurate recall and lead to inflated estimations. This highlights a developmental paradox in which cognitive growth does not necessarily translate into improved self-monitoring of movement behaviours.

Overall, these results call into question the validity of using the PAQ-A as a stand-alone measure of MVPA in adolescents, particularly when precise behavioural estimates are required. While the tool shows acceptable internal consistency and test–retest reliability in various populations [33,52], its criterion validity appears insufficient for high-stakes or intervention studies without objective corroboration. Researchers and practitioners should consider combining self-reports with device-based methods or applying statistical correction models to account for systematic overestimation, especially in female and older adolescent subgroups.

Future research should aim to refine culturally adapted versions of the PAQ-A and explore machine learning or hybrid models that integrate subjective and objective inputs. Additionally, educational interventions targeting metacognitive skills related to activity awareness may enhance adolescents’ ability to report physical activity more accurately. These findings reinforce the broader recommendation to triangulate data sources when assessing movement behaviours in youth populations.

### 4.5. Strengths

Strengths of this study include the combined objective assessment of sleep, sedentary time, and physical activity using 24/7 wrist accelerometry, the inclusion of multiple cognitive domains (IQ, attention, visual memory), and the focus on adolescents from a Central/Eastern European setting, for which there is limited prior evidence. The study also provides a direct comparison between self-reported physical activity (PAQ-A) and device-based MVPA.

### 4.6. Limitations

This study presents several limitations that should be acknowledged. Firstly, the cross-sectional design precludes any causal inferences regarding the relationship between 24 h movement behaviours and cognitive performance. Secondly, the relatively small and region-specific sample may limit generalisability, particularly to broader adolescent populations with differing socioenvironmental contexts. Thirdly, while the use of both objective (accelerometry) and subjective (PAQ-A) methods strengthens ecological validity, self-reported data were subject to response bias and overestimation, especially among older adolescents and females. Additionally, despite employing validated cognitive tests, these tools may not capture the full spectrum of executive functioning, processing speed, or working memory. Finally, the limited statistical power reduced the sensitivity to detect small-to-moderate effects, especially in stratified or post hoc comparisons (e.g., dual adherence to MVPA and sleep guidelines).

## 5. Conclusions

This study adds to emerging evidence on 24 h movement behaviours and cognition in adolescents from a Central/Eastern European context. In this convenience sample, adherence to movement guidelines was low, and we observed small, domain-specific associations, primarily with sleep characteristics (notably sleep timing and deep sleep duration), with selected cognitive outcomes. Given the modest sample size, the large number of tested associations, and the cross-sectional design, the present findings should be interpreted as exploratory and hypothesis-generating, rather than definitive evidence that partial guideline compliance improves fluid intelligence, attention, or memory. The comparison of self-reported physical activity (PAQ-A) with accelerometer-derived MVPA indicated discrepancies that may contribute to measurement bias and should be considered when interpreting behavioural surveillance data. Overall, the results highlight the potential relevance of sleep-related dimensions within the 24 h movement framework and support the need for region-specific monitoring of adolescent movement behaviours and sleep in Central and Eastern Europe. Future research should prioritise larger, adequately powered longitudinal studies and targeted interventions to test whether changes in sleep timing and the accumulation of structured physical activity translate into meaningful cognitive outcomes, while carefully addressing residual confounding and multiple testing. A testable hypothesis is that adolescents who increase structured MVPA (e.g., accumulated in ≥10 min bouts or through organised sport participation) while maintaining adequate sleep duration and earlier sleep timing will show subsequent improvements in attention and working memory in longitudinal designs.

## Figures and Tables

**Table 1:** Sample characteristics and adherence to 24 h movement behaviour guidelines by sex and age group.

Variable		Total (*n* = 82)	Boys (*n* = 32)	Girls (*n* = 50)	15 y (*n* = 12)	16 y (*n* = 32)	17 y (*n* = 24)	18–19 y (*n* = 14)
MVPA (min/day)	Median IQR	32.9 (15.1–49.6)	18.9 (9.1–40.5)	37.7 (22.9–54.1)	43.0 (23.0–57.5)	27.8 (12.6–45.0)	36.2 (19.5–54.0)	26.9 (16.5–42.2)
Sleep duration	Median IQR	433.2 (384.4–487.2)	453.0 (352.1–528.1)	429.2 (404.6–474.6)	435.9 (403.9–465.7)	423.6 (375.6–480.3)	445.0 (390.1–508.9)	443.7 (382.4–499.7)
Sedentary time (min/day)	Median IQR	652.6 (530.4–706.5)	587.7 (196.9–686.9)	666.4 (603.0–706.9)	655.1 (583.1–710.3)	660.2 (442.9–709.3)	628.6 (572.7–687.5)	658.6 (367.3–724.6)
MVPA (≥60 min/day)	%	11.0	3.1	10.0	0.0	15.6	12.5	7.1
Short sleep	%	39.0	40.6	38.0	33.3	46.9	33.3	35.7
Adequate sleep	%	48.8	37.5	56.0	58.3	40.6	54.2	50.0
Long sleep	%	12.2	21.9	6.0	8.3	12.5	12.5	14.3

Note: Values are presented as medians with interquartile ranges (IQRs). MVPA adherence: ≥60 min/day of moderate-to-vigorous physical activity. Sleep categories followed [34]: short sleep ≤ 420 min/night; adequate sleep 421–599 min/night; long sleep ≥600 min/night. Percentages indicate the proportion of participants in each subgroup who meet the respective criteria based on valid accelerometer-derived data.

**Table 2:** Moderation analysis (sleep × MVPA) predicting cognitive outcomes (robust HC3 standard errors).

Outcome	Predictor	B	Robust SE (HC3)	95% CI	*p*	ΔR^2^ (Interaction Added)
IQ	Sleep (h)	1.000	0.791	(−0.551, 2.551)	0.206	
IQ	MVPA (per 10 min)	−0.284	0.741	(−1.737, 1.168)	0.701	
IQ	Sleep × MVPA	0.283	0.297	(−0.300, 0.865)	0.342	0.014
Attention	Sleep (h)	−0.159	1.963	(−4.006, 3.688)	0.935	
Attention	MVPA (per 10 min)	0.639	1.091	(−1.499, 2.777)	0.558	
Attention	Sleep × MVPA	0.107	0.603	(−1.075, 1.290)	0.859	0.001
Memory	Sleep (h)	0.302	0.424	(−0.530, 1.134)	0.477	
Memory	MVPA (per 10 min)	−0.057	0.309	(−0.663, 0.549)	0.853	
Memory	Sleep × MVPA	0.001	0.114	(−0.222, 0.224)	0.991	0.000

Note: Sleep and MVPA were mean-centred prior to computing the interaction term; models adjusted for age and sex; robust HC3 standard errors.

## Data Availability

The raw data supporting the conclusions of this article will be made available by the authors on request.

## Supplementary Materials

The following supporting information can be downloaded at: https://www.mdpi.com/article/10.3390/healthcare14030360/s1, Figure S1: Distributions of daily movement behaviours; Figure S2: Spearman correlations between movement behaviours and cognitive outcomes. Rows represent sleep and activity variables; columns represent IQ, attention, and memory scores. Only significant correlations are colour-coded (red = positive, blue = negative); non-significant correlations (*p* ≥ 0.05) appear with a white background. Each cell displays the correlation coefficient (ρ) and *p*-value; Table S1: Sex differences in sedentary time and physical activity patterns.

## Abbreviations

The following abbreviations are used in this manuscript:

24-HMBs	24 h movement behaviours
ADHD	attention-deficit/hyperactivity disorder
IQ	intelligence quotient
IQR	interquartile range
LPA	light physical activity
MPA	moderate physical activity
MVPA	moderate-to-vigorous physical activity
NDDs	neurodevelopmental disorders
PA	physical activity
PAQ-A	Physical Activity Questionnaire for Adolescents
SB	sedentary behaviour
SD	standard deviation
TIP	Test of Intellectual Potential
VEGA	Scientific Grant Agency of the Ministry of Education, Science, Research and Sport of the Slovak Republic and the Slovak Academy of Sciences
