# Supplementary material for: Associations Between 24 h Movement Behaviours and Cognitive Abilities in Slovak Adolescents: A Cross-Sectional Study
Source: Healthcare (Basel). 2026 Jan 30;14(3):360. doi: 10.3390/healthcare14030360 (PMC12897161; doi:10.3390/healthcare14030360)
Supplement: Supplementary file 1 [file healthcare-14-00360-s001.zip › healthcare-4040678-supplementary.pdf]

Supplementary Materials

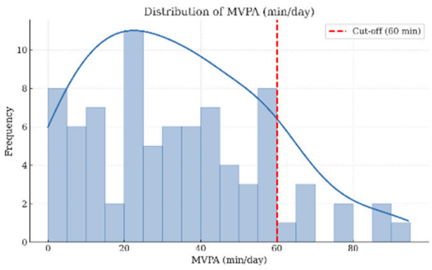

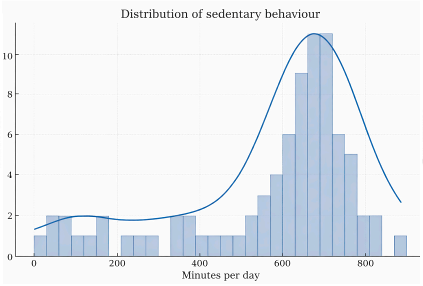

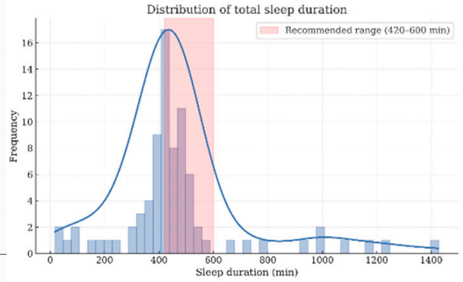

(a) **(b)** **(c)**

Supplementary Figure S1. Distributions of daily movement behaviours.

Supplementary Figure S1 presents the distributions of (a) MVPA (min/day), (b) total sedentary time (min/day), and (c) sleep duration (h/night) by sex and age group. Total sedentary time is reported descriptively; no guideline-based time cut-off is applied, therefore no threshold line is displayed in panel (b).

Variable	Boys / *Median (IQR)*	Girls / *Median (IQR)*	U	p	r
**Sedentary behavior 0–1 min bouts**	60.0 (6.6–79.3)	82.3 (66.1–113.2)	454.0	0.001	0.363
**Sedentary behavior 10–30 min bouts**	85.3 (38.8–125.5)	121.1 (85.4–142.0)	531.0	0.011	0.282
**Sedentary behavior 1–10 min bouts**	103.5 (32.2–128.9)	145.0 (117.2–169.5)	396.5	0.001	0.423
**Total sedentary time (min/day)**	587.7 (196.9–686.9)	666.4 (603.0–706.9)	565.0	0.026	0.246
**LPA 0–1 min bouts**	64.0 (11.0–87.8)	89.4 (67.8–111.7)	461.0	0.001	0.355
**LPA 10–30 min bouts**	27.6 (9.0–42.2)	41.4 (29.9–55.6)	538.5	0.013	0.274
**LPA 1–10 min bouts**	84.2 (25.0–102.6)	121.5 (90.7–140.3)	404.0	0.001	0.415
**LPA total (min/day)**	218.6 (64.7–280.8)	291.2 (248.7–321.6)	453.0	0.001	0.364
**MPA 0–1 min bouts**	6.8 (2.1–12.4)	11.7 (9.5–20.8)	515.0	0.007	0.299
**MPA total (min/day)**	18.8 (7.2–37.3)	37.0 (21.9–51.3)	500.0	0.004	0.314
**MVPA 5–10 min bouts**	0.3 (0.0–4.3)	3.8 (1.1–6.4)	487.0	0.003	0.331
**MVPA total (min/day)**	18.9 (9.1–40.5)	37.7 (22.9–54.1)	506.0	0.005	0.308
**Top 60-min (most active 60-min)**	126.7 (69.7–165.9)	158.5 (133.9–194.3)	498.0	0.004	0.316
**Top 30-min (most active 30-min)**	175.2 (135.6–218.5)	218.0 (177.6–262.0)	497.0	0.004	0.318
**Top 5h value (most active 5 hours)**	54.2 (36.9–64.1)	67.5 (54.6–82.1)	535.0	0.012	0.278
**Top 10h value (most active 10 hours)**	46.4 (28.3–55.1)	55.9 (45.9–65.0)	455.0	0.025	0.256

Supplementary Table S1. Sex differences in sedentary time and physical activity patterns

Note: Medians and interquartile ranges (IQR) are reported separately for boys and girls. Group differences were tested using the Mann–Whitney U test. Effect sizes (r) were derived from the standardized test statistic and interpreted as small (r ≈ 0.1), moderate (r ≈ 0.3), and large (r ≥ 0.5). Only movement behaviour variables with statistically significant sex differences ($p < 0.05$) are presented.

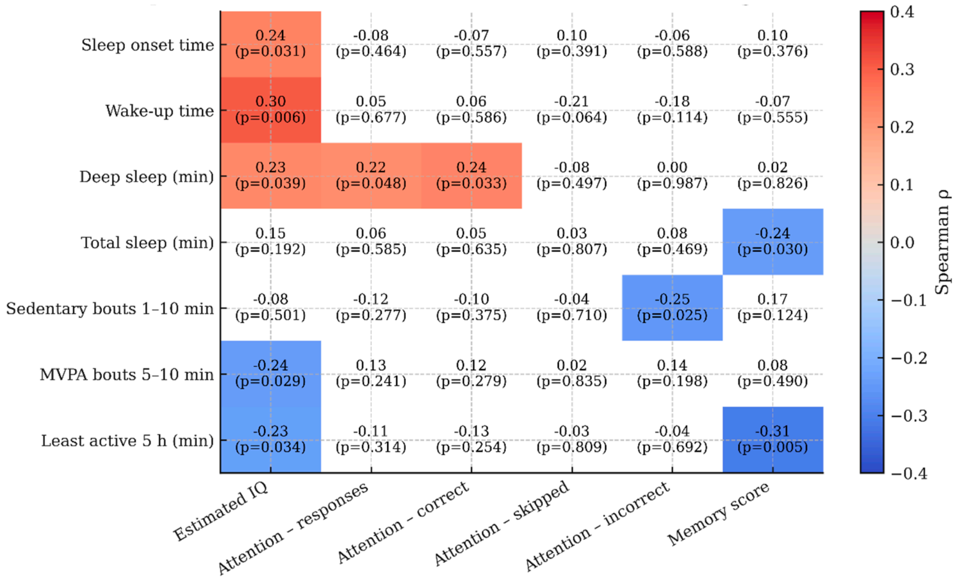

Supplementary Figure S2. Spearman correlations between movement behaviours and cognitive outcomes. Rows represent sleep and activity variables; columns represent IQ, attention, and memory scores. Only significant correlations are colour-coded (red = positive, blue = negative); non-significant correlations ($p \geqslant 0.05$) appear with a white background. Each cell displays the correlation coefficient (ρ) and p-value.
